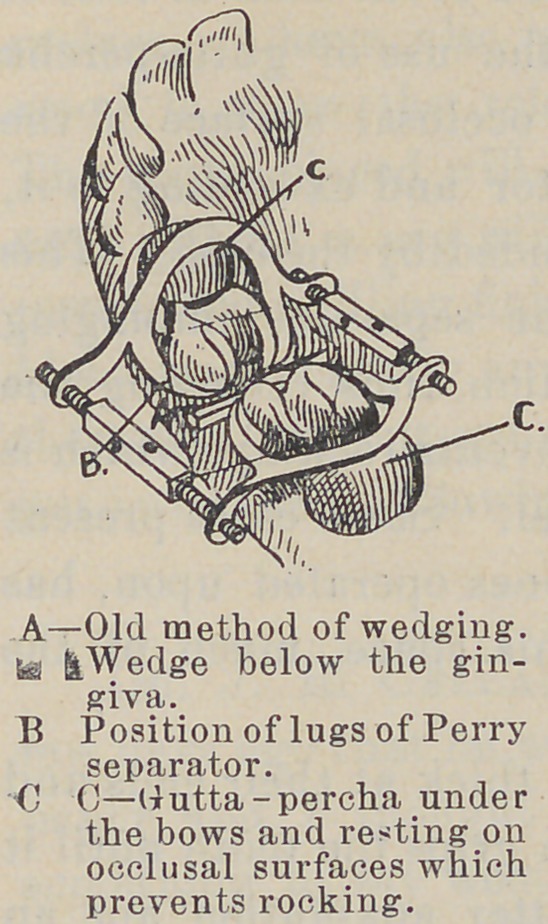# Separators

**Published:** 1895-02

**Authors:** Herny Barnes

**Affiliations:** Cleveland, O.


### Separators.

BY HERNY BARNES, D.D.S., CLEVELAND, O.

Read before the Ohio State Dental Society, December, 1894.

Nature, in infinite wisdom, has so shaped and moulded the teeth as to best serve the purpose for which they were intended. Commencing with the cuspids and including the molars, a solid phalanx is presented, which is well calculated to stand the strain of violent mastication, while preserving to each tooth an individual motion, we have, as a general shape, a convex distal surface and a flattened, or concave and convex mesial surface.

The ideal filling, as to form, has been and is the aim of the conscientious operator. The methods employed have been varied; the materials used have been many; but whatever the method employed or material used, all have agreed that, to restore nature's contour sufficient space must be had to enable the work to be carried on without hindrance and also to afford space for final finishing.

We shall confine our discussion of this subject to the methods in use during the last twenty-five years, and, whatever condemnation we may find, it is not of that which is past, but is confined to the practice of the past, made obsolete by the better of the present.

Previous to the use of cohesive gold and the rubber-dam, the Arthur method was much used. It consisted of grinding or filing the proximate surfaces and leaving V-shaped spaces with a shoulder at the cervix. So unscientific was this procedure that when cohesive gold came into use, it was speedily abandoned. Next came the wedge of wood, which served a long and cruel apprenticeship, we say cruel, because as used by many of the best operators it was merciless, often driven far below the gingiva, it entirely obliterated the gum septum and furnished a nucleus for many chronic diseases. The objections urged against the wooden wedge may also be urged against the rubber, when used as a wedge. Many teeth were operated upon while yet inflamed from the pressure of the rubber, and the nervous condition of the patient under such treatment was such as to preclude the doing of good work. That many good operations were performed is not denied, but that more failures were not noted is the wonder.

Cotton as a separator was then introduced, and while not open to the same objection as wood or rubber, is still quite objectionable on the ground of destroying gum tissue, which should be preserved if the best results are expected.

Dr. Bonwill has explained his method of separating by the use of gutta-percha, by preparing all the cavities in the mouth and then filling from tooth to tooth with gutta-percha and the same left for a number of months. This method may answer in a few cases, but when urged as a practice, is open to serious objections. We must not forget that all practitioners have not the full control of patients as has Dr. Bonwill, so that, while the practice in his hands may produce good results, and no doubt does, it might prove disastrous if generally practiced.

A—Old method of wedging.
A Wedge below the gingiva.
B Position of lugs of Perry separator.
C C—Gutta-percha under the bows and resting on occlusal surfaces which prevents rocking.

A new thought has crystallized and we have what are known as the mechanical separators, invented by Perry and others. We are quite well aware that they are not perfect, but they are far in advance of anything hitherto produced when considered in the light of results. In this light we propose to discuss them.

Many there are who can see no good in them, but we shall try, as best we may, to bring out their good points and endeavor to promote their use among this class of dentists. The objection usually raised, is that the lugs, so press upon the tooth tissue as to mar or check it. This is the strongest objection yet raised and is worthy of consideration.

No instrument will work itself. There must be a controlling mind behind it, and if we expect it to fit equally well any and all cases, we shall find ourselves mistaken. All teeth are not made in the same mold, for we have them long, short, broad and narrow and of complex form. This being true, it must follow that no instrument, however well devised, can be considered universal in use. We must, therefore, resort to expedients and may use any one or a combination of methods in any given case. For example: The Perry separator may be used to obtain immediate space, but the teeth being of such shape as to cause the separator to be dislodged during the operation of filling, may necessitate the use of a wedge of wood to maintain the separation after the removal of the mechanical separator, or, the teeth having been separated by the use of cotton, the mechanical separator may be applied to preserve space and to distribute the force of the mallet blow during the operation. The models here presented will explain the uses of the Perry separator which has given the best results in our hands.

As previously stated, all teeth are not of the same size or shape, so that the lugs of a separator would encroach upon the gingiva more in the case of a short crowned tooth than in that of a longer. This difficulty is obviated by the use of gutta-percha or modelling compound, placed on the occlusal surface of the teeth, underneath the bow of the separator and extending to it, more or less material being used as demanded by the case. This serves a double purpose; it prevents the separator impinging upon the gum and also steadies it in position, thus removing the objection previously raised, in that it prevents rocking, which is the prime cause of the checking of enamel. Some cases present in which a tooth, mesial or distal to the ones operated upon, has been lost, if gutta-percha is placed in this space, much of the pain will be avoided.

We find the lugs of the separator too thick at their ends and our first care is to grind the surface which grips the tooth until it is rather sharp, which permits of a better adaptation and an easier application.

Advantages of the mechanical separator: First, immediate separation may be had in a majority of cases presented. Second, the gum septum is not injured by the pressure of a foreign substance upon it. Third, after an operation has been finished to the point of filling, a few turns of the screw will afford sufficient space for final finishing, which preserves the natural contour so much to be desired, upon its removal the teeth fall back to their points of contact. Fourth, the full matrix may be done away with and in its place a narrow band may be inserted at the cervix and held in place by the lugs of the separator, thus affording the best possible provision for the restoration of full contour. We have now used the Perry separator for a number of years and the results produced warrant its continuance.

We cannot close this paper without a protest against what is known as "The Universal Separator," for this reason, that it is open to very serious objections when used on bicuspids and molars, especially as the lugs on one side are drawn into the proximate space and thus impinge upon the gum tissue, and, in the case of cavities extending to or beyond the cervix, they are in the way and may check frail walls and margins.

The model which I here present will show the Perry separator adjusted. It is the separator designed for the bicuspids and molars. I have also placed in the proximate space the peg of wood to show the relative position of the separators and peg. The peg so placed will destroy the gum tissue. The separator used without a peg will preserve it so that a cavity may be prepared and a filling finished and polished with the loss of little blood and scarcely any injury to the gum tissue. You notice there has been cut out a second molar and gutta-percha has been put in its place, allowing distribution of the mallet blow.

DISCUSSION.

Dr. J. R. Callahan, Cincinnati: Dr. Barnes wrote me a few days ago that he would like me to open the discussion on his paper and I thought I had an easy task, for I thought I had something to say about separators, but when I saw the paper I found that he had said all that I wanted to say and perhaps put it in better language than I could. There is nothing to say except about the slight inflammation that follows the use of cotton or wooden wedges, or anything of that kind. I advocated them for some time and I know I ruined some front teeth by the use of hard wood wedges.

The rubber also causes as much irritation as the wooden wedge and perhaps the cotton is best. So far as filling the teeth with gutta-percha and allowing it to remain for months, I question whether that is good treatment.

He says, too, that no instrument will work itself. How true that is. An instrument is set aside because we don't generally study it up. Many of my friends in the dental profession say they don't like the Perry separator. I don't understand how it is other than because they haven't studied them, or have used inferior separators and have laid them aside as not good. I think no dentist who becomes familiar with them would do without them. You know how difficult it is if you get started to making a particular finish; if you get started in and have a small space it is more than likely you will spoil the contour, whereas if you have a separator, another turn will make the finish.

As to the universal separator, I will say I prefer the Perry.

I have some separators which I would like to give away to somebody I have some spite against. If any one who has not tried this method of separating teeth would take one or two of these separators and use that one or two in a few cases until they get the hang of it and learn the superiority to the use of gutta-percha and such other methods, they would soon have a full set of the instruments.

Dr. Frank Hunter, Cincinnati, stated that he had no experience in mechanical separation, but said that Dr. Heise had a varied fund of knowledge on the subject—it was difficult to extract, and stated that he would like to introduce and hear from him.

Dr. Heise, stated that the subject was covered so thoroughly by the paper that he had nothing to say about it; that he had used the separator for a number of years in the same manner as Dr. Barnes and he could only add his word of recommendation in the use of it.

Dr. Grant Mitchell, Canton: I use the Perry separators and could't keep house without them. The manner in which they keep the rubber out of the way and the facility with which one can get at his work make them remarkable instruments. When here last winter I was talking with Dr. Sillito in reference to separators, and where one has time to separate the teeth he showed a simple device that struck me forcibly and I have used it frequently. I don't know that I can illustrate it perfectly, but I will try. (The doctor then illustrated from the blackboard.)

That represents the set of teeth. Dr. Sillito uses a very fine linen tape. He takes a piece of the tape and passes it between the teeth. Suppose I want to separate those central incisors. I pass the tape between the teeth and tie the knot around and drive it in between them and the force of the knot being drawn between will exercise a mechanical influence there—the swelling of the fiber will make a separation. If there is a large cavity it is difficult to tie that knot. In cases of that kind I pass the knot between the teeth and put a piece of cotton in to fill the cavity and then pull the knot. The credit of that is all due to Dr. Sillito. There is no separator like the Perry.

Dr. C. R. Butler, Cleveland: I have nothing special unless to add something in the way of referring to this tape. It is a very nice thing I know from experience. There is another mode which was suggested a little time ago by Dr. Palmer, of Syracuse. It is a peculiar kind of fiber, very fine, that is used by anglers as a fish line and it has great strength. You can draw it between the teeth and it is surprising how the stiffest tooth will be moved by that fine twine. I was surprised when he presented it and looking at the size of it I could't see how it could have such power, so that the stiffest tooth can be separated a considerable space with very little soreness. I think Dr. Mitchell stated the tying in of this tape and putting in cotton if there was a large cavity was an idea presented by Dr. Sillito. That may be true, but there are a great many that profess to have used the same mode as well as Dr. Sillito. This twine—you can put it between the incisors, bicuspids and even molars and string it through by the gum and tie special knots in it. It won't slip out because the knots prevent it and there is so little bulk it won't interfere with closing the teeth. If there is a cavity between them, put a little cotton in before tying it tight. You can move your teeth apart pretty readily, within a day or two, and excite very little soreness and it dosen't slip down as the rubber does on those stiff cases where you want to open them without an immediate wedging.

I will say a little in regard to the advantage of gaining space. It is imperceptible to the patient. While packing gold there is a good deal said about the use of the band matrix, but should you slip a little piece of steel through, and you could put a little piece of wood there to keep it from slipping down, and if it is a wide space you can put a piece of wood down here or you can bring the steel up to the cavity, or the adjustable crib, by driving the gold in packing out against the steel (indicating) in using it not as a means to hold the gold in the cavity but to pack the gold as if there was no crib there, it will enable you to let it expand against it. You extend the space so you can get a contour in your gold enabling you to do the polishing and to finish right away, and you can finish it whether you have the Perry separator or not.

Dr. Grant Mitchell, Canton: In difficult cases where we want a good deal of space and the cavity is inaccessible, it is difficult to use a Perry separator to gain space. After I have gained space by the tape I will frequently use the Perry separator to hold it, but if it is a case that won't take very long I don't hesitate to put in a piece of wood.

Dr. Grant Mollyneaux, Cincinnati, says that he don't know anything about separators; that he remembered when Dr. Sillito mentioned the method suggested by Dr. Mitchell but did not know that Dr. Sillito claimed it was original; that Dr. Austin Dunn, of Chicago, presented it to the American Dental Association some six or seven years ago. That he never heard anybody dispute his originality in that direction.

Dr. W. B. Ames, Chicago: It gives me a great deal of pleasure to meet with the dentists of my native State, and while I don't have as much occasion to separate teeth as a great many, I make use of the Perry separator. I also have a separator made by Ivory, of Philadelphia, and I use it in as many cases as the Perry.

I want to speak of the method that Dr. Bonwill has spoken of. I don't know whether you have seen the results of such a case. I happened to treat a case directly from Dr. Bonwill's hand. A young girl from Philadelphia had some work done by me. In going down to Pliladelphia and getting into some trouble she was taken by the principal of the school to Dr. Bonwill and he adopted this method, and for some reason she didn't return to him. He began the filling with gutta-percha he had put there for a temporary purpose. As a young child she had a slight defect in the arrangement of the anterior teeth above and below. This gutta-percha had expanded and forced apart the bicuspids and molars so that there was more space than anyone cared for, to make an operation, and there was a marked protrusion of the front teeth above and below which will remain for life. After various operations the space is still there. The separation was very effectual but very disastrous.

Dr. H. A. Smith, Cincinnati: I am very much interested in the subject of separating teeth. I have heard this discussed by such men as Dr. Crouse, of Chicago, and other dentists, and they differ in their method. Dr. Crouse insists he must get a large space between the molars. Another will insist he should get little space. There must be a happy medium. It is important that we should have space sufficient to make a good operation and give a proper contour to the tooth. That depends upon the characteristics both of the patient and the tooth. If we have good teeth and not a very permanent foundation, we might resort to the mediate, and if short teeth it would be better to resort to the immediate method. What method you should use depends on the dentine. I don't separate teeth as much as many persons. Perhaps I don't do as much good to my patients. So far as the gutta-percha method is concerned, I think it is admissible. I think the large spaces that have been spoken of show that, judiciously used, it is a good method.

A disaster that comes from undue separation is the result produced upon the periosteum. We attribute this to other causes frequently. In that admirable book that is issued from the press by Dr. Bodecker, he calls particular attention in his allusions to periodontitis, to its being caused by inflammation made by the use of wedges, especially by mechanical separators. I think many times periodontitis is produced by these forcible methods that we trace to other causes.

I suppose that I have gone through all the methods. I don't know that I have tried this particular one. It is simply a method for retaining it in position. The cotton fiber expands immensely. It is a very gradual and very excellent way. My one experience has been I would rather trust a gradual separation than a forcible separation. You who have had experience in colleges, know how heroically students use separators. They want a large space and they are instructed they must have a large space before they can do well. They use the Perry separator or the universal.

I like one point of the paper, that we must have caution behind it, as to what we shall use and how long we shall use it.

I was very much pleased with the latter part of the paper which I had the pleasure of reading.

Dr. A. F. Emminger, Columbus: It would seem that Dr.

Perry had set things up in this society for his separator. I have not been touched by Dr. Perry, but I think his separators are good things if judiciously used. They can be applied so easily that they have been abused many times. We don't need as much space as we sometimes make. I have used them in the anterior teeth and also in the bicuspids for getting a slight separation, enough to allow for finishing. I have an appliance that is a small straight steel instrument or inverted wedge, very thin, about an inch and a half from the end of the instrument, as thin as these ribbon polishing strips, German silver. I slip that between the teeth and drawing it instead of pushing, it draws the teeth apart as much as necessary without much pain, and after putting a plug between the teeth, drawing the separator back, remove it, and you can gain space very quickly and without any irritation. I don't know whose instrument it is or who invented it. I think these rapid mechanical separators are good in their place if properly used, but they are abused many times, and the separators are not at fault, it is the power behind.

Dr. L. E. Custer, Dayton: I think Dr. Emminger has called attention to one good point that ought to be made use of, and that is that pulling the wedge through serves better than pushing one in.

Dr. Arnold, Columbus: I have used this instrument. It is very useful inasmuch as you don't need to separate your teeth before you begin the operation. We can do a great deal of the work without separation. You only need space for finishing. If you use the instrument described by Dr. Emminger, it is all you need. It is a tapering instrument. Pass in the thin portion and gradually pull through.

Dr. J. Taft: This is a subject interesting to every dentist in performing his operations upon the natural tooth, especially in filling. It is one the principal of which should be well understood by every one. You often hear of rapid separation and again others speak of gradual separation. Some advocate one and some the other. Some use both, discriminating, applying them to the cases they think best adapted to the mode of separation. I think the latter is the proper method. It is not best in all cases to occupy several days in making the separation; not best to make a gradual separation in many cases. In many instances teeth separated in this way become fixed in their new position and either do not return to their former position or become diseased or affected in some way. Both of these results have followed from an indiscriminate gradual separation. I think there is less likelihood of danger from an immediate and prompt separation.

Why do they remain in their new position? When pressure is exerted on a tooth to remove it from its position, a double action is set up—a process of removing from one side and filling in on the other, and if there is nothing to bring the back tooth it is likely to retain the man position. I used to feel a good deal of chargin at these things occurring when I used the gradual separation, as I did formerly.

The prompt separation made by the separators is, in a great many cases, the very best—better on many occounts. In the first place the operation can be accomplished perhaps at one sitting, whereas if pressure were applied, a number of days elapse before it would be accomplished, and in many cases where gradual pressure is applied, soreness would be the result to a considerable degree, inflammation set up, and that sometimes would not pass away for considerable time. I have seen that soreness remain for days after the separation was accomplished before the operation could be well tolerated for the pain. That, of course, is an objectionable state of things.

A prompt separation operates upon the expansive tissue about the tooth. Separated gradually, these would perfectly adapt themselves to the change without bruising the tooth or rupturing the vessels, and the gum tissues are enabled to sustain the pressure. If rapid pressure is used there may be a bruising of the periosteum to such an extent that injury will result afterwards. If separation was made gradually, injury of that sort would not occur.

Then again, in applying the mechanical separators, in many cases the teeth may be separated rapidly and thus be injured by the rapid separation, and moving the separator slowly the blood will flow out of the capillaries and vessels when the pressure is made and the tissue yield without the laceration of any tissue. If the separators are moved rapidly, injury may result by bruising or rupturing the small vessels.

In regard to the appliance used, it doesn't make very much difference except as a matter of convenience, as the principle is the same. It is to bring the pressure to bear between the two teeth to give the desired space for the operation and the separator serves its purpose very well—either the Perry or the Ivory separator, which I believe is called the Universal. I use the Ivory separator perhaps more than the Perry. I have used the Perry separator since it was introduced, but I have used the Ivory perhaps quite as often used the Perry. It is operated by a screw which can be turned slowly to make the pressure gradual so it will not be objectionable to the patient, and he can move it gradually as the operation proceeds. It seems to me that one will make the separation more gradual with that instrument than with most other. The danger with most mechanical separators is too rapid movement.

There are some teeth so firmly fixed that much force is necessary to move them, and it sometimes seems difficult to move them with any force. In such a case I make a space by cutting the tooth—not by cutting off the whole side of the tooth.

The age of the patient ought to be taken into account, as well as the susceptibility of the teeth and parts about them. You find some persons that offer firm, solid bony tissue that will not yield under any reasonable pressure. Sometimes the soft tissues are more resistant than in other cases. Sometimes the tissues will yield to the pressure readily. There need be no excuse for ignorance on these points. If one has his patient in hands for a number of years he should know the susceptibility of the patient —how much can be endured by the patient, and how much can be borne without injuring the tissue.

In regard to tape and things of that kind put between the teeth, they may be used in many cases advantageously. A gradual separation can be accomplished in a little while. Place and draw it between the teeth, putting it so it will make the pressure before tying the knot, tying it between the teeth, and you make a separation very well, but it takes more time than by the Perry or Ivory separator.

I can heartily recommend the Ivory separator. I have no interest in it except an interest in using it. I have used the Perry separators and like them. They are more likely to get cranky than the Ivory, and I have had the screw to become turned more than it ought to be, and it becomes rigid, and is hard to move. I have occasionally had that difficulty, and I know others have, but nothing of the kind could occur with the Ivory separator. I make separation enough to begin work in the cavity, turning this a little at a time so that the patient hardly knows it.

Dr. F. E. Battershell, New Philadelphia: I have used several of the mechanical separators, and when the teeth were closely packed together, in using mechanical separators, there is danger of absorption of the process and a rupture of the vessels at the apex of root. The teeth may become twisted by too rapid separation. Now in such cases it should be done by a slow process, by means of tapes or some other means of that kind. Some slight absorption must take place, but these teeth can be separated without harm, and it has been my observation that the only thing that is safe in such cases is a slow process.

Dr. H. A. Smith: Just a word with reference to the principle of all this. If we separate the anterior teeth—those of a single root, how is it accomplished? It must be by the elongation of both teeth, and a very slight elongation will give you space. If it is persistent, it will give you a larger separation. If you have the double root teeth, then immediate separation is not necessary, and if you get separation it must be by a process of absorption.

Dr. W. H. Todd, Columbus: I have been using the Perry separators for some time, and since using them I find that I separate the teeth less than I did before. In preparing a cavity you often find it is not necessary to separate the teeth as much as you need in the first place. After excavating it we put the rubber dam on, we find we have room to fill the cavity and we still have room to finish the filling.

Dr. Henry Barnes, Cleveland: I rise to apologize. I didn't know there was such unanimity of sentiment in respect to the Perry separator. I thought I would encounter considerable opposition, but most of the men who object to the Perry separator are outside of the Ohio Dental Society. Agrees with all Dr. Taft said up to the point where he speaks of the Perry separator and in favor of the Ivory. In the Ivory separator you have two points of contact, and so far as the separator getting cranky is concerned, there is no separator that will get quite so cranky as the Ivory separator, due to the unequal shape of the teeth on the lingual and labial surfaces. With the Ivory separator you bring your points together and the separator is liable to a movement back and forth, and by that movement you produce pain. In the Perry separator, as illustrated on model, there are three points of contact on each tooth. The lugs grip the tooth at the buccal and lingual angles, which the gutta-percha placed under the bow on the occlusal surface makes the third contact — thus affording steadiness during separation. When you mallet upon them you are not driving the teeth against the cushion, and yon are not getting up an irritation as you are under the old method.

I realize there is more than one way of killing a pig. You may stick him in the neck and he is dead. You may hit him in the head and break his leg and otherwise maltreat him, you don't deny the pig is dead, but it is not the kind of a dead pig you have in the other case. The value of a Perry separator or any other is in the use and not in its abuse.

In regard to the instrument spoken of by Dr. Emminger, in my hands, it is the most painful instrument I have ever used for the separation of teeth.

If I understand Dr. Taft rightly in regard to the Perry separator getting cranky, it it where he has turned one of the bars a little more than he should have done, without turning the other side. If you turn it gradually and don't take it as you would a crow-bar to lift a house, but take hold of it, turn it very gently and bring it around to the points of contact on each side, it is drawn up easily and you have no impinging upon the gum. Every turn of the Ivory separator brings a single point against the tooth and upon the gum tissue. and more than that, it is in the way.

I have used about everything that has been spoken of except the method of Dr. Butler. I don't know anything about that.

## Figures and Tables

**Figure f1:**